# Effects of a Social Media–Based Mind-Body Intervention Embedded With Acupressure and Mindfulness for Stress Reduction Among Family Caregivers of Frail Older Adults: Pilot Randomized Controlled Trial

**DOI:** 10.2196/42861

**Published:** 2023-02-20

**Authors:** Yufang Sui, Patrick Pui Kin Kor, Mengli Li, Jingjing Wang

**Affiliations:** 1 School of Nursing The Hong Kong Polytechnic University Hong Kong Hong Kong; 2 School of Nursing and Health Zhengzhou University Zhengzhou China

**Keywords:** mind-body intervention, acupressure, mindfulness meditation, social media

## Abstract

**Background:**

Family caregivers of frail older adults experience high levels of stress. Mind-body interventions (MBIs) focused on caregiver stress are often limited in teaching approaches, difficult to practice, and costly. A social media–based MBI embedded with mindfulness meditation (MM) and self-administered acupressure (SA) may be effective for family caregivers, offer greater usability, and lead to greater adherence.

**Objective:**

The aim of this study was to test the feasibility and preliminary effects of a social media–based MBI embedded with MM and SA on family caregivers of frail older adults and to investigate the preliminary effects of the intervention using a pilot randomized controlled trial.

**Methods:**

A 2-arm randomized controlled trial design was adopted. Family caregivers of frail older adults (n=64) were randomized into either the intervention group (n=32), receiving 8 weeks of social media–based MM and SA, or the control group (n=32), receiving brief education on caregiving for people with frailty. The primary outcome (caregiver stress) and secondary outcomes (caregiver burden, sleep quality, and mindfulness awareness and attention) were measured using a web-based survey at baseline (T0), immediately after the intervention (T1), and at the 3-month follow-up (T2).

**Results:**

The feasibility of the intervention was established with a high attendance rate (87.5%), high usability score (79), and low attrition rate (1.6%). The generalized estimating equation results showed that participants in the intervention group at T1 and T2 experienced a significant improvement in stress reduction (*P*=.02 and *P*=.04, respectively), sleep quality (*P*=.004 and *P*=.01, respectively), and mindful awareness and attention (*P*=.006 and *P*=.02, respectively) compared with the control group. There were no substantial improvements in caregiver burden at T1 and T2 (*P*=.59 and *P*=.47, respectively). A focus group session conducted after the intervention had 5 themes: impact on the family caregivers, difficulty in practicing the intervention, the strength of the program, the limitations of the program, and perception of the intervention.

**Conclusions:**

The findings support the feasibility and preliminary effects of social media–based MBI embedded with acupressure and MM on reducing stress among family caregivers of frail older people and enhancing sleep quality and mindfulness levels. A future study with a larger and more diverse sample is proposed to evaluate the longer-term effects and generalizability of the intervention.

**Trial Registration:**

Chinese Clinical Trial Registry ChiCTR2100049507; http://www.chictr.org.cn/showproj.aspx?proj=128031

## Introduction

### Frailty and Family Caregiver Stress

Frailty is associated with a wide range of negative health outcomes, such as a higher risk of death, disability, hospitalization, and higher medical expenditures [1], all of which impose a heavy burden on families and society [2]. Nearly one-fourth of individuals aged >50 years were identified as frail, as determined using a frailty index [3], and this proportion increases with age [4]. As most frail older adults want to live at home with their families, they are increasingly seeking support and health-related services from home- and community-based services, which imposes a substantial burden on their caregivers [5]. Informal caregivers of frail older adults are nonprofessionals (usually family members and friends) who take up the duties and responsibilities for caring for older persons, ranging from providing physical aid to emotional support and making decisions related to their daily activities [6,7]. Caregivers of frail older adults need to take on heavy work in providing most caregiving tasks at home, such as providing assistance with activities of daily living, financial support, and supervision [8]. This demanding role could pose a threat to the health of family caregivers, leading to different comorbidities, such as insomnia and cardiovascular diseases [5]. In addition to threats to physical health, family caregivers of frail older adults often experience emotional struggles, burnout, or compassion fatigue, amplifying the stress resulting from caregiving [9]. Most primary caregivers of frail older adults have nowhere to release their pressure because 52.8% of them work alone without the involvement of a second caregiver [10]. Therefore, most primary informal caregivers of frail older people feel stressed because of the high emotional, financial, and physical demands of the work of caring [11] and might experience some psychological and physiological symptoms caused by caregiver stress, such as depression, anxiety, cognitive disturbance, insomnia, aches and pains, headaches, dizziness, muscle tension, etc [12,13]. These symptoms will eventually reduce their quality of life compared with that of the general population [14].

### Mind-Body Intervention

Mind-body interventions (MBIs) encompass a variety of treatments, such as yoga, meditation, acupressure, tai chi, and qigong, which focus on the effects within the mind, body, and health. Studies on MBIs point to potential advantages in reducing psychological pressure and improving quality of life [15]. They enhance the mind’s positive impact on the body [16] and are suitable for family caregivers because MBIs can be practiced by the individual acting alone and are free from the stigma attached to psychological treatment [17]. Mindfulness meditation (MM) is a form of meditation; its psychological mechanism is to expand the individual’s attention in a nonjudgmental manner [18]. The shift in attention during positive reappraisal and stress reduction may liberate practitioners from the source of stress [19,20]. There is growing evidence that mindfulness can improve caregivers’ perceived stress and caregiving burden; control negative thoughts in caregiver as well as anxiety and depressive symptoms, with a medium to large effect size; and improve insomnia [21-23]. The 2 most common evidence-based mindfulness programs, namely “Mindfulness-based Stress Reduction” (MBSR) and “Mindfulness-based Cognitive Therapy,” use mindfulness as a component. However, these 2 mindfulness programs usually require intensive training and a long duration (eg, an 8-week mindfulness program) to improve health-related symptoms [24-26]. They induce a high attrition rate and a longer onset time for improving physical symptoms compared with other nonpharmacological interventions such as acupressure that have a shorter onset time [27,28]. To address some acute stress-related symptoms, the mindfulness intervention protocol could be strengthened by incorporating other therapeutic components with a shorter therapeutic onset time.

### Acupressure

Traditional Chinese medicine (TCM) provides several nonpharmacological interventions for health maintenance and promotion. Acupressure, a technique based on the meridian theory of TCM, is a simpler and easier way to stimulate acupoints using parts of the human body unlike acupuncture that requires use of needles. According to TCM theories, the targeted stimulation of acupoints provides a sense of well-being, leading to the opening of the meridians and balancing of Qi, promoting health by restoring the smooth flow of qi energy, and by sending a signal to the body. Its physiological mechanism for reducing stress is based on deactivating signals from acupoints and meridians to the amygdala, hippocampus, and other brain regions responsible for the heightened effect of anxiety and on producing endogenous opioids, increasing the production of serotonin and reducing the stress hormone cortisol [29]. It can relieve physical symptoms relatively quickly. After 15 minutes of treatment, a significant decrease in stress, fatigue, pain, heart rate, and respiration in patients was observed, and more than half of the participants experienced stress relief for at least 1 to 4 hours [30]. Moreover, acupressure can be administered by individuals themselves after appropriate training and is a low-cost, safe, and flexible method [31,32]. Self-administered acupressure (SA) has been proven safe and has medium effects on reducing stress in family caregivers [33-35]. However, SA only concentrates on stress-related physiological symptoms such as pain, fatigue, and inappetence [30,36] and does not address the need for caregivers to cope with stress and their psychological needs. Previous studies have indicated that auricular-plaster, a type of acupressure, had a larger effect on stress reduction when combined with MM, which indicates that MM could be combined with SA to achieve additional effects on stress reduction among the caregivers of frail older people [37]. Combining MM and SA would be an innovative approach to reducing stress from psychological and physiological pathways and may induce better results than a single MBI.

### The Conceptual Model for Combining MM and Acupressure

**Figure 1:**
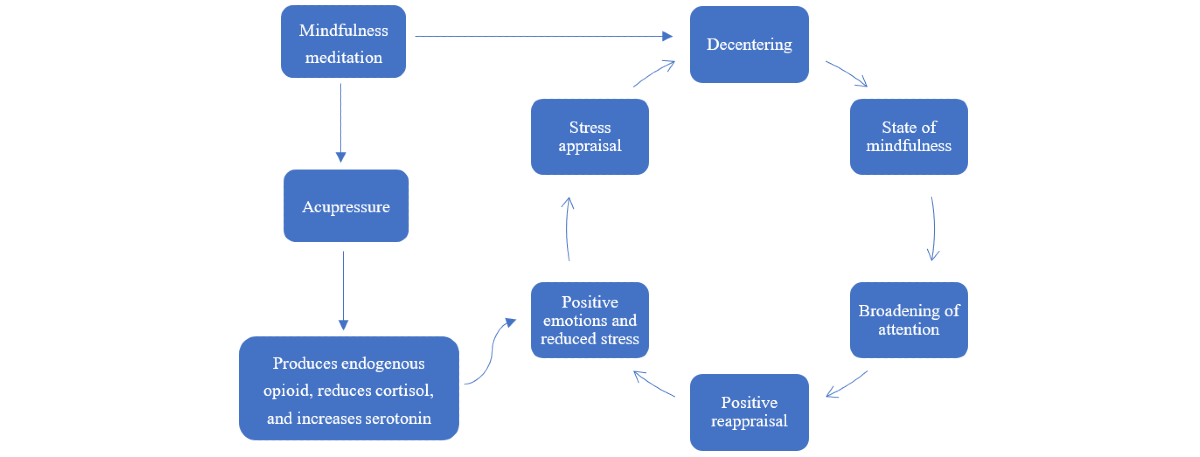
Mindful and acupressure stress-coping model.

According to the mindful stress-coping model [20], the repeated practice of mindfulness will help people generate positive and optimistic perceptions and accumulate positive emotions, leading to an upward spiral of a sense of positivity. In this study, MM and acupressure acted separately on the psychological and physiological aspects of caregiver stress [19,20]. MM induced positive reappraisals, emotions, and reduced stress, whereas acupressure produced the corresponding chemicals to physiologically improve stress. The potential stress reduction mechanism of this combination was demonstrated from both psychological and physiological perspectives using a model (Figure 1). The combination of MM and acupressure may enable the respective strengths of each to be used to enhance their effectiveness, shorten the onset of the intervention, and reduce the adverse effects of the prolonged practice of a single intervention.

### Social Media Platform

In recent years, social media has been increasingly used worldwide to provide health services, health education, and even health interventions, which alters the types and tempos of interactions between individuals and health care providers [38,39]. Studies have shown that caregivers also benefit from health education and training provided through social media platforms [40]. The most popular social media platform in China is WeChat, which has >902 million daily users [41]. One of the most valuable functions of WeChat, called the “WeChat group,” has a capacity of up to 500 people and can be used to make multiperson video calls and share text, images, voice, videos, and rich media messages [42]. The widespread use of WeChat offers a promising and cost-effective medium for supporting health [43]. It has been well accepted by caregivers for improving their ability to provide care, for reducing the burden of providing care, and for changing their health behaviors [44,45]. Thus, it will be a great channel for teaching and training SA and MM on the web when traditional face-to-face training is constrained by geographical barriers or scheduling conflicts, especially during the COVID-19 pandemic.

As is known from the literature review, no research has been conducted that combines acupressure and MM. All complex MBIs using MM as a component are delivered face-to-face rather than self-administered, and there is no standard technique for selecting acupoints or standard duration for reducing caregiver stress. In view of these research gaps, this study aimed to develop an evidence-based MBI that is embedded with MM and SA and delivered via WeChat to relieve the stress of family caregivers of frail older adults. The importance of this research is that it provides culturally appropriate stress reduction interventions for family caregivers of frail older people and improves the accessibility and usability of interventions using electronic platforms, laying the foundation for this complex MBI to be better implemented in the future.

### Objectives

This study aimed to test the feasibility and preliminary effects of a social media–based MBI embedded with MM and SA on the family caregivers of frail older adults and to investigate the preliminary effects of the intervention for further larger-scale randomized controlled trial (RCT) by testing the following hypotheses: (1) this intervention is feasible for the family caregivers of frail older people; (2) the stress of the caregivers would be reduced after the intervention at T1 and the 3-month follow-up at T2 (primary outcome); and (3) caregivers would experience improvements in sleep quality, caregiver burden, and mindful attention and awareness after the intervention at T1 and at the 3-month follow-up at T2 (secondary outcomes).

## Methods

### Study Design

**Figure 2:**
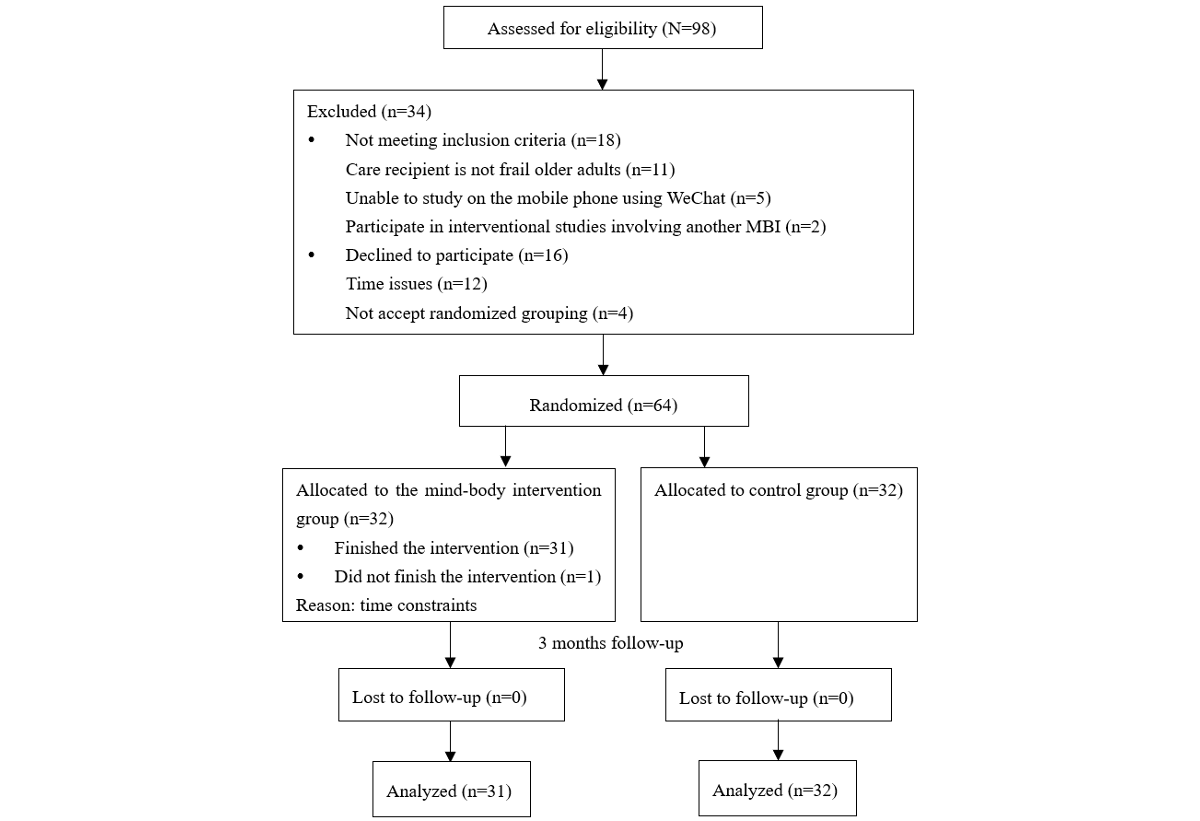
Flow diagram of this study. MBI: mind-body intervention.

A prospective, single-blinded, parallel-group pilot RCT design was adopted (Figure 2). The family caregivers of frail older adults were randomized into either the intervention group receiving the social media–based MBI embedded with MM and SA or the control group receiving brief education on older adult care and usual care.

### Ethics Approval

This trial was registered with the Chinese Clinical Trial Registry (ChiCTR2100049507) and approved by the Human Subjects Ethics Sub-committee of The Hong Kong Polytechnic University (HSEARS 20210608004).

### Participants and Settings

Convenience sampling was adopted from April 20 to July 1, 2021, to recruit community-dwelling family caregivers of frail older adults from the physical examination center of a grade III–level hospital, which receives 1.72 million annual outpatient visits in Henan province, China. Caregivers were included if they were (1) aged ≥18 years and could understand Chinese, (2) the primary family caregiver of frail older adults (≥60 years) with frailty (a score on the simple frailty questionnaire [FRAIL] of ≥3), (3) providing unpaid caregiving (helping frail older adults with activities of daily life) for at least 14 hours per week [33], and (4) using WeChat and able to study on mobile phones. Caregivers were excluded if they (1) had contraindications to acupressure or MM (eg, suspected fractures, tumors, tuberculosis, severe heart and lung disease, pregnancy, and infected skin and wounds in the selected region); (2) had an acute psychological problem; and (3) had participated in interventional studies involving acupressure, acupuncture, MM, or other MBI (eg, Tai chi and yoga) within the past 6 months.

### Sample Size

Previous studies recommended a sample size of 12 per group for a pilot RCT to produce at least moderate effects in the main study [46]. Considering a dropout rate of 20%, 30 participants were required. In the end, 64 participants were recruited.

### Randomization, Allocation, and Blinding

Those who met the eligibility criteria and provided informed consent were randomly allocated in a 1:1 ratio to one of the following groups: (1) a social media–based MBI embedded with SA and MM and (2) brief social media–based education by block randomization and computer-generated random numbers with a block size of 4. An independent researcher, who was not involved in collecting or entering the data, delivered the interventions. After baseline data collection, participants received notice of their group allocation through personal phone calls and messages from the independent research assistant. The group allocation lists were concealed from other researchers, the TCM and MM professionals, and the outcome assessors.

### Interventions

#### The MBI Group

The Medical Research Council Framework for the development and evaluation of complex interventions guided this MBI design process. First, available information related to SA and MM intervention protocols was identified from the literature. For intervention parameters involving complex interventions on meditation, the duration of effective intervention programs ranged from 4 to 12 weeks, with 8 weeks as the median [37,47,48]. Five times per week and 15-20 minutes of meditation were the most frequently used intervention frequencies [37,47,48]. The techniques for operating MBIs usually included exercises on thoughts and feelings, awareness of breath, and a body scan [49]. No adverse events were reported. The 15-minute MM training and practice audio for this study was adapted from the first 2 weeks of the 8-week MBSR program created by Jon Kabat-Zinn, which contains the above components and is of appropriate duration. The MM was delivered by a certified MBSR instructor from the mindfulness center of the University of Massachusetts.

The intervention parameters of the acupressure were derived from previous studies [33,50-55]. The number of acupoints used was 1, 3, 5, and 9, with 5 as the median number. The total number of acupuncture points used was 14. The 4 acupoints, Baihui (GV20), Neiguan (PC6), Shenmen (HT7), and Taichong (LV3), which are easily visible and located, were selected multiple times. The duration of the effective intervention programs was 2-16 weeks, with 4 weeks being used most often. To consider the length of time for practicing MM and to further ensure the dose, a median of 8 weeks was the most appropriate. Given the different acupoints and parameters related to acupressure ingredients and implementation technique specifications, it is essential to provide a standard SA protocol in complex interventions to relieve stress. We convened a panel of Chinese acupressure experts to conduct a multiround consensus survey using the Delphi technique. Furthermore, 6 TCM experts were invited to participate in the Delphi method. They were instructed to select each item by integrating the research evidence with their knowledge and clinical experience [56]. The criteria used to identify specialists included (1) a history of publishing on acupressure, (2) a minimum of 5 years of postregistration experience and specialization in acupressure, or (3) a recommendation from participants who met the above criteria. These experts were not invited to participate in the other parts of the study. We sent each expert an invitation letter and questionnaire. The questionnaire items were drawn from studies published in the last 5 years related to acupressure for stress relief; all the acupoints and parameters mentioned were listed in the questionnaire. The consultative questions focused on the selection of intervention ingredients, including four domains: (1) therapeutic principles, (2) acupoint selection and combination, (3) operation techniques, and (4) possible adverse effects and solutions. Comment boxes were also attached to provide the experts the opportunity to share their thoughts. After the first Delphi round, the experts’ suggestions were analyzed, summarized, and collated into new items that were added to the second round of the Delphi questionnaires. The content assessment form used 3-option questions, namely “Agree,” “Neutral,” and “Disagree,” to measure the attitude of the experts toward each item [57]. The expert panel’s authority (Cr), bias of judgment (Ca), and familiarity (Cs) with the participant were used to evaluate the reliability of the questionnaire. The mean Cr value of this Delphi process was 0.85, which suggests that the expert panel’s authoritative degree was adequate for gaining the desired opinions. The items in the second Delphi round that received a rating with a consensus of >80% are listed in Table 1. The techniques used to identify acupoints and operation of acupressure were based on complete agreement from the expert panel and literature reviews published in the past 6 years (Table 1).

**Table 1:** Agreement on the acupressure protocol by the expert panel in the second Delphi round (N=6).

Items			Agreement, n (%)				
			Agree		Neutral		Disagree
**Selection of acupoints**							
	Baihui (GV20)	6 (100)		0 (0)		0 (0)	
	Neiguan (PC6)	5 (83)		1 (17)		0 (0)	
	Shenmen (HT7)	6 (100)		0 (0)		0 (0)	
	Zusanli (ST36)	5 (83)		1 (17)		0 (0)	
	Taichong (LV3)	5 (83)		1 (17)		0 (0)	
**Operation techniques**							
	Once a day	5 (83)		1 (17)		0 (0)	
	Duration is 3 minutes per acupoint	5 (83)		1 (17)		0 (0)	
	Press 1-2 minutes per point and rest 2-3 minutes after all points are pressed, for a total of 2-3 rounds of circulation	5 (83)		1 (17)		0 (0)	
	Pressure should be sufficient to evoke a “de qi” sensation (ie, soreness, numbness, distention, and heaviness)	5 (83)		1 (17)		0 (0)	
**Adverse reactions and solutions**							
	Unbearable pain	5 (83)		0 (0)		0 (0)	
	Numbness and senselessness	5 (83)		0 (0)		0 (0)	
	Stop acupressure immediately when adverse reactions occur	5 (83)		0 (0)		0 (0)	
	Timely feedback to the TCM^a^ therapist for further treatment	5 (83)		0 (0)		0 (0)	

^a^TCM: traditional Chinese medicine.

The intervention group received 9 education sessions, mainly delivered via the social media platform (first, second, third, and ninth sessions) by a TCM practitioner and mindfulness therapist, as well as self-directed web-based learning (fourth, fifth, sixth, seventh, and eighth sessions) with phone support from a registered nurse. In the first 2 sessions, MBI training consisted of 40 minutes per week, with 20 minutes of MM and 20 minutes of SA. A communication group was set up for the participants to share their learning experiences and ask questions on a social media platform.

In the third week, TCM and mindfulness specialists helped the participants to review their knowledge and gave them web-based video tests on SA and a web-based audio test on MM to ensure that they had the skills for daily practice. The web-based examination of SA was conducted by a TCM specialist using the multiplayer video function so that each participant could see other participants in real time. The 15-16 participants were divided into 2 small groups of 7-8 people. The members of one group first demonstrated the methods used to locate acupoints and their SA skills, and the members of the other group evaluated their performance. These 2 small groups then exchanged tasks. The TCM expert kept close records of each participant’s performance and evaluated all participants during the entire process. Participants who could correctly locate and press all acupoints were regarded as qualified, whereas those who failed to meet this standard were reassessed after the review until they passed the examination. TCM specialists confirmed the sensation by asking questions of the participants and observing their behavior during the web-based examination to ensure that they had mastered the operation techniques. The mindfulness specialist conducted an MM web-based test through a web-based audio examination by asking the participants questions that tested their understanding of MM. The caregivers were given booklets and videos for self-directed learning through a social media platform.

From week 4 to week 9, each teaching session lasted 40 minutes. MBI were performed once a day, 5 days a week, for 8 weeks. In addition, TCM and MM specialists were available to offer participants additional one-on-one support and guidance via WeChat or phone calls. Details of the intervention process and content are provided in Multimedia Appendix 1.

#### Control Group (Brief Education on Caregiving)

Participants in the control group were provided with brief caregiver education via the social media platform by a nurse experienced in gerontology. The control group was the same size as the intervention group, and their sessions were of the same duration and frequency as those of the intervention group, to control social interaction effects that might affect the measurement of the outcomes. Details of the educational process and content are provided in Multimedia Appendix 2.

### Outcomes and Measurement

Feasibility was defined as 80% completion of the intervention protocol and questionnaires, including daily responses to app-based surveys [58]. Usability was measured through the System Usability Scale, a 10-item scale used to assess the ease and appropriateness of the use of mobile intervention components. Responses were given on a 5-point Likert scale ranging from 1 (strongly disagree) to 5 (strongly agree).

Fidelity checking was conducted each month during the intervention period based on an intervention fidelity checklist that covered the content of each session. The checklist followed the SA protocol and was reviewed by experienced, qualified TCM specialists. The general areas of focus addressed in this pilot study are summarized in Multimedia Appendix 3. All training sessions were audio-recorded and checked against a fidelity checklist by an independent researcher. An acceptable fidelity rate of >90% was adopted [59]. Only 1 TCM and 1 MM specialist delivered all MBI sessions to minimize variations in the implementation of interventions.

Participants’ attendance in weekly training sessions and the duration of their home practice were used to determine the adherence rate and duration of the intervention. Information on their frequency of practice and the duration of their daily practice was collected through the use records of meditation software (Meditation Planet, version 2.1.10; Guangzhou Count Sheep Technology Company Limited) and their acupressure diary. The research assistant collected the adherence data weekly. The author conducted a focus group interview with 12 participants from the intervention group. A semistructured interview guide (Multimedia Appendix 4) was used to identify the impacts on the family caregivers, their difficulty in practicing the intervention, their strengths and limitations, and their perceptions of the program and intervention.

### Primary and Secondary Outcomes

The primary outcome in this study was the participants’ stress level, which was measured at baseline (T0), immediately after the intervention (T1), and at the 3-month follow-up (T2). The Chinese version of the Perceived Stress Scale (CPSS; CPSS-10) was used to measure the caregivers’ stress levels. The CPSS-10 is a 5-point Likert scale with 10 items (Cronbach α=.91, intraclass correlation coefficient=0.69), with 0=never to 4=very often [60]. The total score ranged from 0 to 57, with higher scores denoting a higher level of perceived stress. The secondary outcomes were the participants’ sleep quality, caregiver burden, and mindful attention and awareness, which were measured at T0, T1, and T2. The Chinese version of the Pittsburgh Sleep Quality Index (CPSQI), a 4-point Likert scale with 19 items (Cronbach α=.83), was used to assess 7 components of sleep quality: the use of sleep medication, sleep quality, sleep latency, sleep duration, sleep efficiency, sleep disturbances, and daytime dysfunction. Higher scores indicated greater levels of insomnia. The CPSQI has demonstrated satisfactory internal consistency and test-retest reliability in Chinese populations [61]. Caregiver burden was measured using the Chinese version of the Zarit Burden Interview, which is a 5-point Likert scale with 22 items (Cronbach α=.87) used to address the perceived impact of the act of providing care on the physical health, emotional health, social activities, and financial situation of the caregiver [62]. The Chinese version of the Mindful Attention and Awareness Scale, a 15-item single-dimension measure of mindfulness, measures the frequency of open and receptive attention and awareness of ongoing events and experiences. Response options range from 1 (almost always) to 6 (rarely). Scoring involves calculating the mean performance across items, with higher scores indicating greater mindfulness (Cronbach α=.85) [63]. The Chinese version of the FRAIL, a simple 5-item scale (intraclass correlation coefficient=0.708), was used to assess frailty in older adults [64]. It consists of 5 components: fatigue, resistance, ambulation, illness, and weight loss. FRAIL scores range from 0 to 5 (ie, 1 point for each component), with 0 representing robust, 1 to 2 prefrail, and 3 to 5 frail statuses [65].

### Data Analysis

Descriptive statistics were used for the quantitative data of baseline characteristics and the recruitment rate, attrition rate, frequency, and consistency of participants practicing the MBI. An independent-sample *t* test (2-tailed) was used to compare sociodemographic and baseline outcome variables between the 2 study groups. Generalized estimating equations (GEEs) were adopted to examine the outcomes of the study between the intervention and control groups across the 3 time points (T0, T1, and T2). The dependent variables were the total scores of caregivers’ health outcomes (stress, caregiver burden, sleep quality, and mindful awareness and attention) in the GEE analysis. The independent variables were the group, time points, and group × time interaction. Missing data were estimated by using multiple imputations in the GEE model. SPSS (version 23.0; IBM Macintosh) was used to analyze the data. An intention-to-treat analysis was performed.

The focus group interview was conducted by a senior research nurse asking the participants about the complexity of the teaching content, the rationality of the teaching method, the difficulties they faced during participation, the help that they needed, their satisfaction with the project, the impact of the intervention on their life, health, and other aspects; their comparisons of acupressure and MM in the intervention; other questions associated with the above aspects; and other comments. The data collected in the interviews were digitally audio-recorded, transcribed verbatim for content analysis by a research assistant, and supervised by the corresponding author. The research assistants read the text repeatedly and then summarized the content, looking for meaningful text and assigning codes, and then sorted the codes into groups to generate themes [66]. When disagreements arose, the authors continued the discussion until consensus was reached.

## Results

### Participant Characteristics

The participant flow diagram is shown in Figure 2. A total of 98 family caregivers showed an interest in joining this study, of which 80 met the sample selection criteria and 64 agreed to participate in the study. Participants were randomly allocated to either the intervention group (n=32) or the control group (n=32) during 2 rounds of participant recruitment.

The demographic characteristics of the family caregivers are presented in Table 2. On average, the participants were aged 47.1 (SD 5.2) years, and the majority (49/64, 77%) were female. Most (44/64, 69%) of the participants were the adult children of frail older adults. There were no substantial differences in the baseline demographics and FRAIL scores between the 2 groups (*P*>.05 for all).

**Table 2:** Demographic data of the participants.

		All (n=64)	Intervention group (n=32)	Control group (n=32)	*P* value	
**Sex, n (%)**						.38
	Male	15 (23)	6 (19)	9 (28)		
	Female	49 (77)	26 (81)	23 (72)		
Age (years), mean (SD)		47.1 (5.2)	46.5 (5.5)	47.7 (4.9)	.38	
**Relationship, n (%)**						.32
	Spouse	7 (11)	2 (6)	5 (16)		
	Parents	44 (69)	22 (69)	22 (69)		
	Siblings	1 (3)	1 (3)	1 (3)		
	Relatives	8 (12)	6 (19)	2 (6)		
	Other	3 (4)	1 (3)	2 (6)		
**Income per month per person, n (%)**						.84
	<US $293.7	6 (9)	3 (9)	3 (9)		
	US $293.7-587.4	39 (61)	19 (60)	20 (62)		
	US $587.4-881.1	19 (30)	10 (31)	9 (28)		
**Education level, n (%)**						.60
	Junior high school degree or below	17 (27)	7 (22)	10 (31)		
	High school or technical school	24 (37)	13 (41)	11 (34)		
	Higher vocational education or junior college	17 (27)	9 (28)	8 (25)		
	Bachelor’s degree or above	6 (9)	3 (9)	3 (9)		
Duration of care (months), mean (SD)		25.6 (11.6)	24.5 (11.9)	25.8 (11.2)	.66	
Duration of care per week (hours), mean (SD)		27.25 (7.5)	26.7 (6.9)	27.8 (8.1)	.55	
Frailty, mean (SD)		3.41 (0.7)	3.34 (0.6)	3.47 (0.8)	.48	

### Feasibility of the Interventions

#### Recruitment

The outpatient department of the hospital was invited to participate in the project. Targeted introductory leaflets were used to recruit participants. Daily public seminars were organized in the waiting area to introduce MM and SA. Potential participants were invited to complete a brief screening using the Chinese version of FRAIL. Eligible participants were then asked to complete a web-based consent form. A total of 80 family caregivers were eligible to participate and 64 agreed to participate. The recruitment rate was 80%.

#### Attendance Rate, Attrition Rate, Acceptability, and Duration of MBI

The mean attendance rate for the live web-based teaching sessions was 87.5% (SD 6.3%) for the intervention group and 89.6% (SD 3.6%) for the control group. There was no significant difference in attendance rates between the 2 groups (*P*=.75). The attrition rate was 2% (1/64), the completion rate of the intervention was 90.6% (1160/1280), and the completion rate of the questionnaires was 98% (63/64). The mean score on the System Usability Scale was 79, higher than the score of 68, which is an indicator of acceptability, indicating that this program’s social media platform was well accepted. Data on the duration and frequency of the practice of mindfulness and acupressure practice are shown in Table 3.

**Table 3:** Duration of practicing mind-body intervention (n=32).

Mind-body intervention				Value
**Self-administered acupressure**				
	Duration (minutes), mean (SD)			76.2 (6.2)
	**Participants, n (%)**			
		<75 minutes	1 (3)	
		75-80 minutes	27 (84)	
		80-85 minutes	4 (13)	
**Mindfulness meditation**				
	Duration (minutes), mean (SD)			83.6 (7.2)
	**Participants, n (%)**			
		<80 minutes	1 (3)	
		80-90 minutes	26 (81)	
		90-100 minutes	5 (15)	

### Preliminary Effects of the MBI

The mean scores of the 2 groups on the CPSS-10, Zarit Burden Interview, CPSQI, and Chinese version of the Mindful Attention and Awareness Scale (at recruitment, immediately after the intervention, and 3 months after the intervention) were compared across time and are summarized in Table 4. Compared with the control group, the participants in the intervention group showed significant improvements in stress, sleep quality, and mindful awareness and attention (*P*=.02, *P*=.004, and *P*=.006, respectively) at T1 and (*P*=.04, *P*=.01, and *P*=.02, respectively) T2. However, there were no substantial improvements in caregiver burden. Caregivers reported no harmful or adverse events.

**Table 4:** Preliminary effect on stress, burden, sleep, and mindful attention and awareness.

Outcomes and time point			Intervention group (n=31), mean (SD)		Control group (n=32), mean (SD)		GEE^a^ analysis, *P* value				
							Group effect		Time effect		Time × group effect
**Stress (CPSS^b^ -10)**											
	T0^c^	24.19 (3.53)		23.59 (3.64)		N/A^d^		N/A		N/A	
	T1^e^	22.32 (3.37)		23.28 (2.99)		.82		.001		.02	
	T2^f^	23.64 (2.95)		23.63 (2.92)		.87		.001		.04	
**Burden (CZBI^g^)**											
	T0	44.94 (7.68)		43.69 (9.25)		N/A		N/A		N/A	
	T1	44.84 (6.41)		43.88 (7.93)		.57		.87		.59	
	T2	45.03 (6.50)		43.66 (8.43)		.53		.99		.47	
**Sleep (CPSQI^h^)**											
	T0	11.77 (1.82)		11.94 (2.08)		N/A		N/A		N/A	
	T1	10.71 (1.60)		12.13 (1.76)		.047		.04		.004	
	T2	11.16 (1.66)		12.16 (1.71)		.03		.096		.01	
**Mindfulness (CMAAS^i^)**											
	T0	63.74 (5.40)		64.66 (5.76)		N/A		N/A		N/A	
	T1	64.97 (5.21)		64.81 (5.60)		.78		＜.001		.006	
	T2	64.13 (5.47)		64.28 (5.27)		.819		＜.001		.02	

^a^GEE: generalized estimating equation.

^b^CPSS: Chinese version of the Perceived Stress Scale.

^c^T0: baseline.

^d^N/A: not applicable.

^e^T1: immediately after the intervention.

^f^T2: 3-month follow-up.

^g^CZBI: Chinese version of the Zarit Burden Interview.

^h^CPSQI: Chinese version of the Pittsburgh Sleep Quality Index.

^i^CMAAS: Chinese version of the Mindful Attention and Awareness Scale.

### The Results of the Focus Group Analysis

Twelve participants aged ranging from 41 to 56 (mean 48.3, SD 5.3) years were invited to participate in the focus group interview. Purposive sampling was adopted by selecting equal proportions of participants with different stress reduction levels (measured using the CPSS-10) after the intervention. More than half (n=7, 58%) were children of frail older people, and one-third (n=4, 33%) were relatives of care recipients. Five themes were identified in the data analysis: impacts on the family caregivers, difficulty in practicing the intervention, strengths of the program, limitations of the program, and perception of the intervention (Table 5).

**Table 5:** Results of the focus group interview.

Themes and categories		Quotations	Participant and page ID
**Impacts on the family caregivers**			
	Improved sleep quality	I fall asleep faster than I used to.	F9^a^-P1^b^-25
	Decreased stress and anxiety	It helped me a lot. I feel less anxious than I used to.	F9-P1-25
	Feelings of relaxation	The acupressure gets rid of all my bad feelings in the body. I felt that MM^c^ could give me complete relaxation.	M1^d^-P1-30 F9-P2-2
**Difficulty in practicing intervention**			
	Acupoints location	I could not locate some acupoints accurately in the beginning, but with the help of the teacher and the booklet, I mastered them.	M4-P1-22
**Strengths of the program**			
	Delivery mode	Web-based teaching is very suitable—mainly because it saves time. It is very convenient as I can listen to the lessons from anywhere.	M2-P1-15 F3-P1-17
**Limitations of the program**			
	Learning atmosphere	Web-based teaching does not fully guarantee a lively classroom atmosphere and is related to self-awareness.	M3-P1-18
**Perception of the intervention**			
	Auxiliary tools	My fingers sometimes get tired of acupressure- so I hope to use some tools in replace of my finger to press the acupoints.	M4-P2-9

^a^F: female participant.

^b^P: page.

^c^MM: mindfulness meditation.

^d^M: male participant.

## Discussion

### Principal Findings

This study used an MBI embedded with SA and MM to reduce psychological and physiological stress levels through a social media platform. The findings from this study suggest that this complex MBI is a feasible and acceptable program for family caregivers, given the high completion rate, attendance rate, and positive feedback collected from the focus group interviews. Substantial improvements in stress, sleep quality, and mindful awareness and attention were observed at T1 and T2 in the intervention group compared with the control group, with no substantial enhancement in caregiver burden.

### Comparison With Prior Work

Although the intervention duration was not the shortest in our study, the retention rate was >90%. Overall, 72% (23/32) of participants attended each program session, with a mean attendance rate of > 85% (SD 4.9%) in both groups. In addition, the participants were very satisfied with our program, as the mean score for satisfaction with the overall program was 9.16 (SD 0.8) out of 10. The attrition rate was lower than that in the study using an SA intervention via the face-to-face mode for stress reduction [33], and it was slightly higher than that in a study using a mobile MM app in working university students [67]. A study has shown that participants can tolerate SA for approximately 15 minutes [33]; there are also reports that participants can meditate for 15 minutes or more each day [37,68]. Retention rate might be affected by the duration of the MBI and the selection of acupoints if they are not easy to position and press. An analysis of 91 comparative studies conducted between years 2000 and 2020 showed that 37 (41%) found web-based teaching to be related to higher learning outcomes and 17 (18%) preferred face-to-face education [69]. The use of a web-based mode of teaching involving a social media platform and a mobile app in this study received much positive feedback in the focus group interview, including its ease of use, the involvement of the learner, and instant responses to questions and concerns.

This study also showed that the MM app (Meditation Planet) could be used to relieve stress and improve sleep quality through the practice of appropriate MM exercises. The results were consistent with those of a study on sleep disturbance associated with improvements in sleep quality, in which an MM app was used for more than 10 minutes per day for 8 weeks [67]. Given that no study has used the practice of MM with this free app, the data in this study have implications for the dissemination of this mobile app at the community level to improve symptoms associated with caregiver stress as a professional and free alternative.

This study found a substantial interaction effect (time × group) on caregivers’ stress, sleep quality, and mindful awareness and attention at T1 and T2, indicating that MBI had a substantial effect on improving caregivers’ stress, sleep quality, and mindful attitude. This finding is similar to that of previous research showing that SA or MM significantly improved participants’ stress level, sleep quality, and mindful attention and awareness. The result on caregiver burden in this study was different from previous studies that showed that a mindfulness-based intervention had substantial effects on caregiver burden [70,71], whereas acupressure did not prove to be significant in reducing caregiver burden. A possible reason for this result on caregiver burden is that MBI did not lead to a reduction in caring tasks. Another possible reason is that there may have been insufficient time to practice MM.

Additional effects on relieving anxiety were also found in the analysis of the focus group interviews on self-reports from the participants. Similarly, a study showed that participants’ anxiety was significantly lower after the use of self-acupressure compared with the results for the enhanced standard care group [72]. Future studies could include in-depth explorations of the effects of MBIs on anxiety and other aspects. As suggested by the interviewees, in the future, the use of assistive gadgets can be discussed with TCM experts for some participants who experience finger tiredness from the extended practice of SA. A 4-arm trial that included meditation with a massage group and a single type of intervention group found no significant differences between groups [47]. Therefore, in a future study, the MBI used in this study could be compared with each of the 2 single components of that intervention, namely SA and MM.

### Strengths and Limitations

This is the first study to use an MBI embedded with SA and MM to reduce psychological and physiological stress levels through a social media platform. In this study, the intervention was developed from the perspective of a combination of the body and mind. The scope of health promotion and stress reduction was more comprehensive than that of a single type of intervention, especially for psychosomatic diseases. A tailor-made intervention protocol with an appropriate number of acupoints, considering factors such as duration, efficacy, relations of compatibility, and other factors, is convenient for participants to recognize and remember. Simplifying the duration and frequency of the intervention also reduced the withdrawal rate and improved participants’ compliance. Moreover, WeChat served as an eHealth platform to provide support and facilitate health exchanges among practitioners and clients, making health services free from geographical and temporal constraints.

First, most participants were women and young adults who had more experience and knowledge of using a social media platform and mobile app. In addition, previous studies indicated that women may have benefited more from mindfulness interventions [73]. A future study with a larger and more diverse sample is needed to evaluate the longer-term effects and generalizability of the intervention. Second, the outcome measures focused only on family caregivers; thus, frail older adults may have been affected by changes in caregivers’ behaviors. The psychological and physiological status of caregivers might also have been influenced by changes in the health status of care recipients. Hence, outcomes related to frailty in older adults should be examined. Third, this study did not measure other mental health outcomes, such as depression, anxiety, and quality of life, as secondary outcomes, and all outcomes were examined using self-reported questionnaires. Future studies should consider more caregiver-related mental health outcomes (eg, suicidal ideation) and physiological data to measure the effectiveness of the intervention. Finally, this study had no no-treatment control group and no longer had a follow-up period, potentially affecting its internal validity.

### Conclusions

The findings of this study suggest that a social media–based MBI embedded with acupressure and mindfulness is a feasible and acceptable program for family caregivers of frail older adults. The intervention program provides caregivers with skills to deal with caregiving stress through a web-based platform. The preliminary results indicated that MBI effectively reduced the stress of family caregivers and improved their sleep quality and mindful awareness and attention. A future study with a larger and more diverse sample is required to evaluate the longer-term effects of the program and increase the generalizability of the study.

## Appendix

Multimedia Appendix 1 The intervention group session content.

Multimedia Appendix 2 The control group education session content.

Multimedia Appendix 3 Key areas of feasibility, fidelity, and possible outcomes.

Multimedia Appendix 4 Semistructured interview guide.

Multimedia Appendix 5 CONSORT e-HEALTH checklist (V 1.6.1).

## Abbreviations

CPSQI: Chinese version of the Pittsburgh Sleep Quality Index
CPSS: Chinese version of the Perceived Stress Scale
FRAIL: simple frailty questionnaire
GEE: generalized estimating equation
MBI: mind-body intervention
MBSR: Mindfulness-based Stress Reduction
MM: mindfulness meditation
RCT: randomized controlled trial
SA: self-administered acupressure
TCM: traditional Chinese medicine

## Data Availability

The data sets generated and analyzed during this study are available from the corresponding author upon reasonable request.
