# Supplementary material for: Effects of a Social Media–Based Mind-Body Intervention Embedded With Acupressure and Mindfulness for Stress Reduction Among Family Caregivers of Frail Older Adults: Pilot Randomized Controlled Trial
Source: JMIR Form Res. 2023 Feb 20;7:e42861. doi: 10.2196/42861 (PMC9989915; doi:10.2196/42861)
Supplement: Multimedia Appendix 1 [file formative_v7i1e42861_app1.docx]

**The intervention group session content**
