# Supplementary material for: Effects of a Social Media–Based Mind-Body Intervention Embedded With Acupressure and Mindfulness for Stress Reduction Among Family Caregivers of Frail Older Adults: Pilot Randomized Controlled Trial
Source: JMIR Form Res. 2023 Feb 20;7:e42861. doi: 10.2196/42861 (PMC9989915; doi:10.2196/42861)
Supplement: Multimedia Appendix 2 [file formative_v7i1e42861_app2.docx]

| **Session** | **Objectives** | **Contents** | **Materials** |
| --- | --- | --- | --- |
| 1 | • Understand and identify frailty | • Overview of frailty | • Teaching video  • Article |
| 2 | • Assessment of Frailty | • Symptoms and outcomes of frailty | • Teaching video  • Article |
| 3 | • Understand physiological changes in older adults | • Physiological changes in older adults | • PowerPoint file  • Teaching video |
| 4 | • Understand psychological changes in older adults | • Psychological changes in older adults | • PowerPoint file  • Teaching video |
| 5 | • Understand the characteristics of Geriatric Nursing | • Characteristics of Geriatric Nursing | • PowerPoint file  • Teaching video |
| 6 | • Manage conflicts in Geriatric Nursing | • Common Conflicts and Handling in Geriatric Nursing | • PowerPoint file  • Teaching video |
| 7 | • Prevent and handle accidents in Geriatric Nursing | • Accident prevention and handling | • PowerPoint file  • Teaching video |
| 8 | • Nursing for common disease | • Nursing of diabetes, coronary heart disease and hypertension | • PowerPoint file  • Teaching video |
| 9 | • Review of education contents | • Review of teaching contents  • Question answering | • Teaching video  • Article  • PowerPoint file |

**The control group education session content**
