# Supplementary material for: Effects of a Social Media–Based Mind-Body Intervention Embedded With Acupressure and Mindfulness for Stress Reduction Among Family Caregivers of Frail Older Adults: Pilot Randomized Controlled Trial
Source: JMIR Form Res. 2023 Feb 20;7:e42861. doi: 10.2196/42861 (PMC9989915; doi:10.2196/42861)
Supplement: Multimedia Appendix 3 [file formative_v7i1e42861_app3.docx]

|  | **Area of Focus** | **Evaluation Method** | **Sample Outcomes** |
| --- | --- | --- | --- |
| **Feasibility** | Acceptability | - Assessed at the start and the end of the intervention to measure the client’ s agreement and collect feedback - System Usability Scale (SUS) for WeChat - Caregiver satisfaction with treatment was assessed at the end of   the study | - Recruitment rate for recruitment capability - Attrition rate - Intent to continue use - Intervention satisfaction - Focus group |
| **Fidelity** | Therapist competence | - Rated through the training video and audio recordings by other therapists | - Focus group |
|  | Adherence | - Session fidelity checklists were developed specifically for a rating in this study - Raters were blind to the treatment plan | - Frequency and times of use of the intervention - The number of sessions completed by caregivers was documented - Focus group |

**Key areas of feasibility, fidelity and possible outcomes.**
