# Supplementary material for: Effects of a Social Media–Based Mind-Body Intervention Embedded With Acupressure and Mindfulness for Stress Reduction Among Family Caregivers of Frail Older Adults: Pilot Randomized Controlled Trial
Source: JMIR Form Res. 2023 Feb 20;7:e42861. doi: 10.2196/42861 (PMC9989915; doi:10.2196/42861)
Supplement: Multimedia Appendix 4 [file formative_v7i1e42861_app4.docx]

Semi-structured Interview Guide

The interview followed the questions as below:

1. Do you find the content easy to understand? Why？
2. Do you think this online, social media-based delivery method is appropriate? Why?
3. Did you face any difficulties in implementing the course? What kind of help did you need?
4. What did you gain from participating in this project?
5. Which of the two interventions, acupressure or meditation, did you find most helpful? Why?
6. Is there anything else you would like to say about the project that I haven’t asked you about?
